# Supplementary material for: Content-rich biological network constructed by mining PubMed abstracts
Source: BMC Bioinformatics. 2004 Oct 8;5:147. doi: 10.1186/1471-2105-5-147 (PMC528731; doi:10.1186/1471-2105-5-147)
Supplement: Additional File 2 — The original results of the above study (non-essential files are deleted to keep the file size under the limit set by BMC bioinformatics). [file 1471-2105-5-147-S2.bz2 › chilibotAdditionalFile2/dip20/277ID1827203E1097/html/ATF3_JUN.html]

 


 **ATF3** and **JUN** 
  
Found 44 abstracts in PubMed, retrieved 20.  
 

 What does Google say? 
 PDF only 
| .edu only 

---

**Interactive relationship** (e.g. stimulation, inhibition, etc)

**Neutral relationship**- We also report the novel finding that a component present in this inducible AP 1  [ **JUN** ]  complex is ATF 3  [ **ATF3** ] .  Ref: 11044097 J Virol, 2000
- Furthermore, DNA binding studies demonstrate that  **ATF3**  binds directly to the AP 1  [ **JUN** ]  site within the cyclin D1 promoter.  Ref: 11375399 J Biol Chem, 2001
- **ATF3**  is a transcription factor belonging to the  **Jun**  Fos family whose mouse homologue TI 241 was isolated, using the differential screening method, from B16 mouse melanoma cells as a blood borne metastasis associated gene.  Ref: 10965025 Jpn J Cancer Res, 2000
- Injury specific expression of activating transcription factor 3  [ **ATF3** ]  in retinal ganglion cells and its colocalized expression with phosphorylated c  **Jun** .  Ref: 10937548 Invest Ophthalmol Vis Sci, 2000
- CONCLUSIONS The transcription factor ATF 3  [ **ATF3** ]  is specifically induced upon optic nerve injury and colocalizes with p c  **Jun**  in surviving ganglion cells.  Ref: 10937548 Invest Ophthalmol Vis Sci, 2000
- Activating transcription factor 3  [ **ATF3** ]   **ATF3**  is a basic leucine zipper protein which is highly homologous to c  **Jun**  dimerization protein 2 JDP2, especially within the threonine proline phosphoacceptor site, Thr 148.  Ref: 12225289 Biochem J, 2002
- Moreover, the results suggest that  **ATF3**  might induce growth factor independence by down regulating a subset of the genes repressed by v  **Jun** .  Ref: 11314051 Oncogene, 2001
- Partial transformation induced by  **ATF3**  was found to be associated with repression of multiple cellular genes that are also down regulated by v  **Jun** , including those coding for the extracellular components fibronectin, decorin, thrombospondin 2, and the pro apoptotic protein Par 4.  Ref: 11314051 Oncogene, 2001
- In the transient expression assay,  **ATF3**  suppressed the p53 gene promoter activity through its specific binding to an atypical AP 1  [ **JUN** ]  element, PF 1 site, in the p53 gene promoter.  Ref: 12161427 J Biol Chem, 2002
- LPS stimulation leads to an induction of ATF 3  [ **ATF3** ]  and JunD factor binding to the CRE AP 1  [ **JUN** ]  site.  Ref: 10760799 Eur J Immunol, 2000
- Gel shift, immunoprecipitation, and cotransfection assays demonstrated that a complex containing ATF2, c  **Jun** , and  **ATF3**  increased binding to the ATF CRE site in the homocysteine treated cells and activated the  **ATF3**  gene expression, while  **ATF3**  appeared to repress its own promoter.  Ref: 10979959 Blood, 2000

**Non-interactive relationship** (e.g. studied together, co-existance, homology, etc.)

- Using in situ hybridization and immunohistochemistry, we compared the expression profile of ATF 3  [ **ATF3** ]  with those of ATF 2 and c  **Jun**  after middle cerebral artery MCA occlusion.  Ref: 12877985 Brain Res Mol Brain Res, 2003
- We conclude that the combination of  **ATF3**  and c  **Jun**  induces the anti apoptotic factor Hsp27, which directly or indirectly activates Akt, and thereby possibly inhibits apoptosis and induces nerve elongation.  Ref: 12832543 J Neurosci, 2003
- Expression of the activating transcription factor 3  [ **ATF3** ]  prevents c  **Jun**  N terminal kinase induced neuronal death by promoting heat shock protein 27 expression and Akt activation.  Ref: 12832543 J Neurosci, 2003
- We have previously shown that homocysteine causes activation of c  **Jun**  NH 2 terminal kinase JNK and activating transcription factor 3  [ **ATF3** ]  liver regenerating factor 1  **ATF3**  LRF1  [ **ATF3** ]  and induces apoptosis in human umbilical vein endothelial cells HUVECs.  Ref: 11726207 Biochem Biophys Res Commun, 2001
- Previous studies in mammalian cells suggested that like other bZip family members e.g.  **Jun**  and Fos,  **ATF3**  might play a role in the control of cell proliferation and participate in oncogenic transformation.  Ref: 11314051 Oncogene, 2001
- Among the 41 statistically significant named overexpressed genes at the 1 hour time point were early growth response 1 EGR1,  **jun**  B proto oncogene  **jun**   [ **JUN** ]  B,  **jun**  D proto oncogene  **jun**   [ **JUN** ]  D, and activating transcription factor 3  [ **ATF3** ]   **ATF3** .  Ref: 12414521 Am J Pathol, 2002
- METHODS The adult rat optic nerve was crushed intraorbitally, and expression profiles of ATF 3  [ **ATF3** ] , ATF 2, and phosphorylated c  **Jun**  p c  **Jun**  were examined by immunohistochemistry and ISH.  Ref: 10937548 Invest Ophthalmol Vis Sci, 2000
- This cluster included genes encoding stress related proteins e.g.  **Jun** ,  **ATF3** , TAB1, and TANK, suggesting that their up regulation may be in response to secondary necrosis.  Ref: 12488458 J Biol Chem, 2003
- Immunoprecipitation analysis and promoter assay for Hsp27 expression suggest that both  **ATF3**  and c  **Jun**  are necessary for transcriptional activation of Hsp27.  Ref: 12832543 J Neurosci, 2003
- cooperative works of ATF 3  [ **ATF3** ]  and c  **Jun**  may be crucial triggers of various transcriptional responses to the ischemic insult.  Ref: 12877985 Brain Res Mol Brain Res, 2003
- IR was found to enhance the expression of c  **Jun**  and, in particular,  **ATF3** , but, in contrast to various other stress stimuli, did not induce the expression of c Fos.  Ref: 12833146 Oncogene, 2003
- RESULTS In response to optic nerve injury, substantial expression of ATF 3  [ **ATF3** ]  as well as that of p c  **Jun**  was observed in the retinal ganglion cells.  Ref: 10937548 Invest Ophthalmol Vis Sci, 2000
- To investigate this putative  **ATF3**  function directly, the rat  **ATF3**  protein was compared with v  **Jun**  for its ability to transform primary cultures of chick embryo fibroblasts CEFs.  Ref: 11314051 Oncogene, 2001
- DOX rapidly activated JNK and c  **Jun**  and induced  **ATF3**  at both mRNA and protein level.  Ref: 12392999 J Mol Cell Cardiol, 2002
- Electrophoretic mobility shift assays identified Nrf2, MafG,  **ATF3** , and  **Jun**  and Fos family members as StRE binding proteins.  Ref: 12453873 Am J Physiol Renal Physiol, 2003
- A higher degree of colocalization was observed for ATF 3  [ **ATF3** ]  and c  **Jun**  than the modest codetection for ATF 2 and c  **Jun** .  Ref: 10937548 Invest Ophthalmol Vis Sci, 2000
- These findings suggest that both ATF 3  [ **ATF3** ]  and c  **Jun**  are crucial to trigger various transcriptional responses and may act synergistically during the survival phase of the optic nerve in the injury model.  Ref: 10937548 Invest Ophthalmol Vis Sci, 2000
- PURPOSE To ascribe activating transcription factor ATF 3  [ **ATF3** ]  as a specifically induced transcription factor after ON injury and to describe its putative role as a modulator of c  **Jun**  transactivation.  Ref: 10937548 Invest Ophthalmol Vis Sci, 2000
- The expression profiles of ATF 3  [ **ATF3** ]  and c  **Jun**  were virtually similar, but c  **Jun**  expression was also observed in other regions of the brain in control rats.  Ref: 12877985 Brain Res Mol Brain Res, 2003
- Like CEFs accumulating v  **Jun** , CEFs accumulating the  **ATF3**  protein displayed a typical, fusiform morphology, associated with an enhanced capacity to grow in medium with reduced amount of serum.  Ref: 11314051 Oncogene, 2001
- **ATF3**  mediated promoter activation is reduced to 1.3 fold and 1.6 fold respectively when the AP 1  [ **JUN** ]  or CRE sites are mutated, and mutation of both sites simultaneously leads to the complete abrogation of promoter activation.  Ref: 11375399 J Biol Chem, 2001
- Overexpression of  **ATF3**  by adenovirus inhibits the mitogen activated kinase kinase kinase 1 MEKK1 c  **Jun**  N Terminal Kinase JNK induced apoptosis and induces neurite elongation via Akt activation in PC12 cells and superior nerve ganglion neurons.  Ref: 12832543 J Neurosci, 2003
- However, in contrast to v  **Jun**  transformed CEFs, the  **ATF3**  overexpressing cells could not promote colony formation from single cells in agar.  Ref: 11314051 Oncogene, 2001
- Northern blot analysis and co transfection experiments demonstrate that overexpression of  **ATF3**  enhances cyclin D1 mRNA expression and activates the cyclin D1 promoter 2.5 fold when activating protein 1 AP 1  [ **JUN** ]  and cyclic AMP response element CRE sites within the promoter are intact.  Ref: 11375399 J Biol Chem, 2001
- Double immunohistochemical labeling revealed concurrent expression of ATF 3  [ **ATF3** ]  and phospho c  **Jun**  in neurons.  Ref: 12877985 Brain Res Mol Brain Res, 2003
- Our results suggest that  **ATF3**  and c  **Jun**  induced Hsp27 expression is a novel survival response in neurons under death stress such as nerve injury.  Ref: 12832543 J Neurosci, 2003
